# Pharmacological inhibition of the ubiquitin-specific protease 8 effectively suppresses glioblastoma cell growth

**DOI:** 10.1515/biol-2022-0562

**Published:** 2023-02-09

**Authors:** Yu Long, Zengchun Hu, Dian Yang, Fuqiang Wang, Chen’ge Zhao, Yang Zhang, Yingqiu Zhang, Hui Ma, Huiyi Lv

**Affiliations:** Department of Pharmacy, The Second Affiliated Hospital, Dalian Medical University, No. 467 Zhongshan Road, Dalian 116000, China; Department of Neurosurgery, The Second Affiliated Hospital, Dalian Medical University, Dalian 116000, China; Institute of Cancer Stem Cell, Dalian Medical University, Dalian 116000, China; Dalian Kexiang Technology Development Co., LTD, No. 467 Zhongshan Road, Dalian 116000, China

**Keywords:** GBM, AURKA, USP8, small molecule inhibitor

## Abstract

Glioblastoma (GBM) is a malignant brain tumor. The purpose of this study is to estimate the potential effects and underlying mechanisms of a ubiquitin-specific protease 8 (USP8) small-molecule inhibitor on the phenotypic characteristics of GBM cells. The growth, migration, invasion, and stemness of GBM LN229 and T98G cells were evaluated by conducting cell proliferation, colony formation, wound healing, transwell, Ki-67 staining, spheroid formation, and ionizing radiation assays, and the results collectively showed the suppressive effects of USP8 inhibition on GBM cells. Furthermore, transcriptomic profiling of GBM cells treated with the USP8 inhibitor deubiquitinase (DUB)-IN-1 revealed significantly altered mRNA expression induced by pharmacological USP8 inhibition, from which we confirmed downregulated Aurora kinase A (AURKA) protein levels using immunoblotting assays. Our findings indicated that the proliferation, invasion, and stemness of LN229 and T98G cells were markedly suppressed by USP8 inhibition. Pharmacological USP8 suppression elicits multiple tumor-inhibitory effects, likely through dysregulating various mRNA expression events, including that of the key cell cycle regulator and oncogenic protein AURKA. Therefore, our observations corroborate the GBM-supportive roles of USP8 and suggest pharmacological USP8 inhibition is a viable therapeutic approach to target GBM. The purpose of this study was to investigate the effect and mechanism of action of the USP8 inhibitor DUB-IN-1 on GBM.

## 1 Introduction

Glioblastoma (GBM) is a high**-**grade subtype of malignant glioma that is categorized as grade IV astrocytoma by the World Health Organization and is often associated with an unfavorable prognosis [1]. Despite advances in the development of multiple therapies against this malignancy, clinical outcomes of patients with GBM have remained unsatisfactory [2,3]. Currently, maximum protection surgical resection, adjuvant radiotherapy, and chemotherapy are the major therapeutic modalities in the clinical management of patients with GBM. Therefore, further investigation on GBM is urgently needed to identify potential therapeutic targets for the development of predictive biomarkers and effective therapies [4].

Regulated protein degradation has been established as a vital controlling factor during tumorigenesis, with related oncogenic proteins considered potential therapeutic targets in the treatment of many cancers [5,6]. Protein ubiquitination is the dominant post-translational modification that mediates protein degradation [7] and involves a wide range of cellular activities, such as apoptosis and cell survival. Protein ubiquitination is a reversible process of post-translational modification, with deubiquitinases (DUBs) catalyzing the release of ubiquitin molecules covalently bound to substrates [8]. Therefore, the deubiquitinase activity of DUBs can counteract the proteasomal or lysosomal degradation of ubiquitinated protein substrates [9]. Ubiquitin-specific proteases (USPs) are the biggest family of DUBs [7], and several members of the USP family have been reported to participate in the initiation and development of human malignancies including GBM.

Ubiquitin-specific protease 8 (USP8), also referred to as UBPY [10], is a deubiquitinating enzyme implicated in tumorigenesis of multiple cancer types, including breast cancer [11] and glioma [12]. Therefore, USP8 has become increasingly considered as a potential anti-cancer therapeutic target. In 2010, DUB-IN-1 (compound 22 d) and a series of its analogs (compounds 22 b–f) were synthesized by Colombo et al. [13], all of which were reported to be potent inhibitors of USP8 deubiquitinating activity. DUB-IN-1 and its analogs inhibited the growth of colon and prostate cancer cells, with IC_50_s ranging 0.5–1.5 µM. Novel USP8 inhibitors were further discovered and exhibited anticancer efficiency, and treatment with the USP8 inhibitor or siRNA targeting USP8 was reported to inhibit HER-3-positive gastric cancer cell growth [14]. Previous studies have shown that the USP8 inhibitor DUBs-IN-2 effectively inhibited the growth of breast cancer cells [11]. In the current study, we evaluated the influence of the USP8 inhibitor, DUB-IN-1, on the phenotypic features of GBM cells *in vitro*. Our findings showed that the pharmacological inhibition of USP8 effectively hindered the proliferation, invasion, and stemness of GBM cells. Transcriptomic profiling of GBM cells treated with the USP8 inhibitor DUB-IN-1 revealed altered expression of multiple genes implicated in tumorigenesis, including the key cell cycle regulator Aurora kinase A (AURKA). Therefore, our results corroborate the oncogenic properties of USP8 in GBM tumorigenesis and advocate it as an achievable anti-cancer target for future development.

## 2 Materials and methods

### 2.1 Cell culture

Human GBM T98G, LN229, and U87MG cell lines were purchased from the American Type Culture Collection (ATCC; Manassas, VA, USA). LN229 cells were cultured in Dulbecco’s modified Eagle’s medium (DMEM; Gibco, USA). U87MG and T98G cells were cultured in Eagle’s Minimum Essential Medium (EMEM; Gibco, USA). All cells were cultured in medium supplemented with 10% fetal bovine serum (FBS; Excel, China) and 1% penicillin/streptomycin (Thermo Fisher Scientific, USA) with 5% CO_2_ at 37℃.

### 2.2 Antibodies and reagents

The antibodies used were mouse anti-GAPDH antibody (Proteintech, 60004-1, 1:5,000, China), mouse anti-Ki-67 antibody (BD Biosciences, 558616, 1:100, USA), and mouse anti-AURKA antibody (Abcam, ab13824, 1:4,000, USA). USP8 inhibitor DUB-IN-1 was purchased from MedChemExpress (MCE, HY-50736, USA).

### 2.3 Cell viability assay

GBM cell proliferation was determined using the 3-[4,5-dimethylthiazol-2-yl]-2,5-diphenyltetrazolium bromide (MTT) assay, as previously described [15]. LN229, U87MG, and T98G cells (4 × 10^3^ of each) were seeded in 96-well plates with 200 µL of medium. The following day, the cells were treated with the USP8 inhibitor DUB-IN-1 or dimethylsulfoxide (DMSO) as a control at various concentrations. Twenty microliters of MTT solution (5 mg/mL) were added to each well to detect cell proliferation after 24 and 48 h of culture, and then, the plates were incubated for 2–4 h at 37°C. The medium was removed, and 150 µL of DMSO (Solarbio, China) was added to each well to dissolve formazan with shaking for 15 min. Absorbance was measured using a spectrometer at 570 and 630 nm.

### 2.4 Colony formation assay

Seeding densities of 1,500 cells/well (T98G) and 2,000 cells/well (LN229) were seeded in six-well plates as previously described [16]. Cells were treated with the USP8 inhibitor DUB-IN-1 or DMSO as a control, and drugs were replenished every 36 h. After 2 weeks of culture, the cells were fixed with methanol (Kermal, China) for 15 min and stained with 0.1% crystal violet for 15 min. The cell colonies were imaged with ChemiDoc XRS + and quantified using ImageJ software.

### 2.5 Ionizing radiation (IR) assay

Seeding densities of 1,500 cells/well (T98G) and 2,000 cells/well (LN229) were seeded in six-well plates as previously described. The following day, cells were treated with the USP8 inhibitor DUB-IN-1 or DMSO as a control and irradiated using an X-RAD 320ix Biological Irradiator (Precision X-ray Inc.) at doses of 2, 4, and 6 Gy in 2 h. The inhibitor was refreshed every 3 d. After 2 weeks of culture, the cells were fixed with methanol (Kermal, China) for 15 min and stained with 0.1% crystal violet for 15 min. The cell colonies were imaged using ChemiDoc XRS + and quantified with the ImageJ software.

### 2.6 Transwell assay

Cell migration and invasion were detected using Transwell chambers (8 µm pore size, Costar). Seeding densities of 4 × 10^4^ cells (LN229 and T98G) were seeded into the upper chamber after treatment with the USP8 inhibitor DUB-IN-1 or DMSO as a control, and the lower chambers contained 500 µL medium with FBS. Following incubation for 24 h at 37°C, the migrated cells were fixed with methanol for 15 min and stained with 0.1% crystal violet for 15 min. The cells were imaged using an inverted microscope (Leica, Germany) and quantified using ImageJ software.

### 2.7 Ki-67 staining assay

The cells were then cultured on sterilized glass coverslips. After treatment with the USP8 inhibitor DUB-IN-1 or DMSO as a control for 24 h, the cells were fixed in 4% paraformaldehyde for 15 min, treated with 0.2% Triton X100 for 5 min, blocked in 2% bovine serum albumin (BSA) for 30 min, incubated with anti-Ki-67 antibody for 30 min, and then incubated with the secondary antibody for 45 min. Coverslips were then mounted on glass slides with 10 µL of Mowiol supplemented with 4′,6-diamidino-2-phenylindole I (DAP; Life Technologies, USA). Using a fluorescent microscope (Leica, Germany) to capture the images, Ki-67 expression was quantified using ImageJ software.

### 2.8 Cell lysis and immunoblotting

Immunoblotting procedures were followed as previously described [17]. After treatment of LN229 and T98G cells with the USP8 inhibitor DUB-IN-1 or DMSO as a control for 24 h, cells were treated with radio immunoprecipitation assay buffer (10 mM Tris–HCl pH 7.5, 1% w/v Triton X-100, 150 mM NaCl, 1% sodium deoxycholate, and 0.1% w/v sodium dodecyl-sulfate [SDS]), centrifuged at 15,000 rpm for 20 min, and analyzed using a Coomassie protein assay kit. The total protein sample were separated using 10% SDS-polyacrylamide gel electrophoresis and then transferred to nitrocellulose membranes (Merck Millipore, USA), blocked with 4% fat-free milk 1 hour at room temperature, and then incubated overnight with the primary antibody at 4℃. The blots were then incubated with secondary antibodies for 1 hour and scanned using a LICOR Odyssey system. Image Studio (version 4.0) software was used to analyze the images.

### 2.9 Spheroid formation assay

A spheroid formation assay was conducted as previously described [18]. Seeding densities of 500 cells/well (T98G and LN229) were seeded in 96-well plates (ultra-low attachment) in the serum-free DMEM-F12 medium supplemented with B27 (2% v/v), basic fibroblast growth factor (20 ng/mL), epidermal growth factor (20 ng/ml), and 1% penicillin/streptomycin. After treatment with the USP8 inhibitor DUB-IN-1 or DMSO as a control for 3 d, the USP8 inhibitor DUB-IN-1 and DMEM-F12 medium were added every two d. After 2 weeks, the images were captured using a phase-contrast microscope (Leica, Germany) on days 7 and 14. The spheroid sizes were quantified using Photoshop (version 2019) software.

### 2.10 Wound healing assay

A 200 µL pipette tip was used to create a wound in a confluent monolayer of cells, as previously described [19]. The cells were cultured in a medium without FBS and treated with the USP8 inhibitor DUB-IN-1 or DMSO as a control. Cell migration was examined using an inverted microscope (Leica, Germany) at 0, 48, and 96 h. The migration distance of the cells was measured using Photoshop (version 2019) software to quantify the wound healing ratio.

### 2.11 RNA preparation and sequencing

After treatment with the USP8 inhibitor DUB-IN-1 (500 nM) or DMSO as a control for 24 h, total RNAs from LN229 and T98G cells in 6 cm dishes were extracted using Trizol reagent (Thermo Fisher, USA). The integrity of the RNA was detected, and first-strand cDNA and second-strand cDNA were synthesized following fragmentation. The library fragments were purified for polymerase chain reaction, and the library quality was determined after purification.

### 2.12 Statistics

All experiments were performed at least thrice. Consequences were expressed as mean ± standard error of the mean. A two-tailed Student’s *t*-test was performed using GraphPad Prism (version 8) software to estimate statistical distinctions between the groups. Statistical significance was set at *P* < 0.05.

## 3 Results

### 3.1 Pharmacological USP8 inhibition suppresses GBM cell proliferation

**Figure 1:**
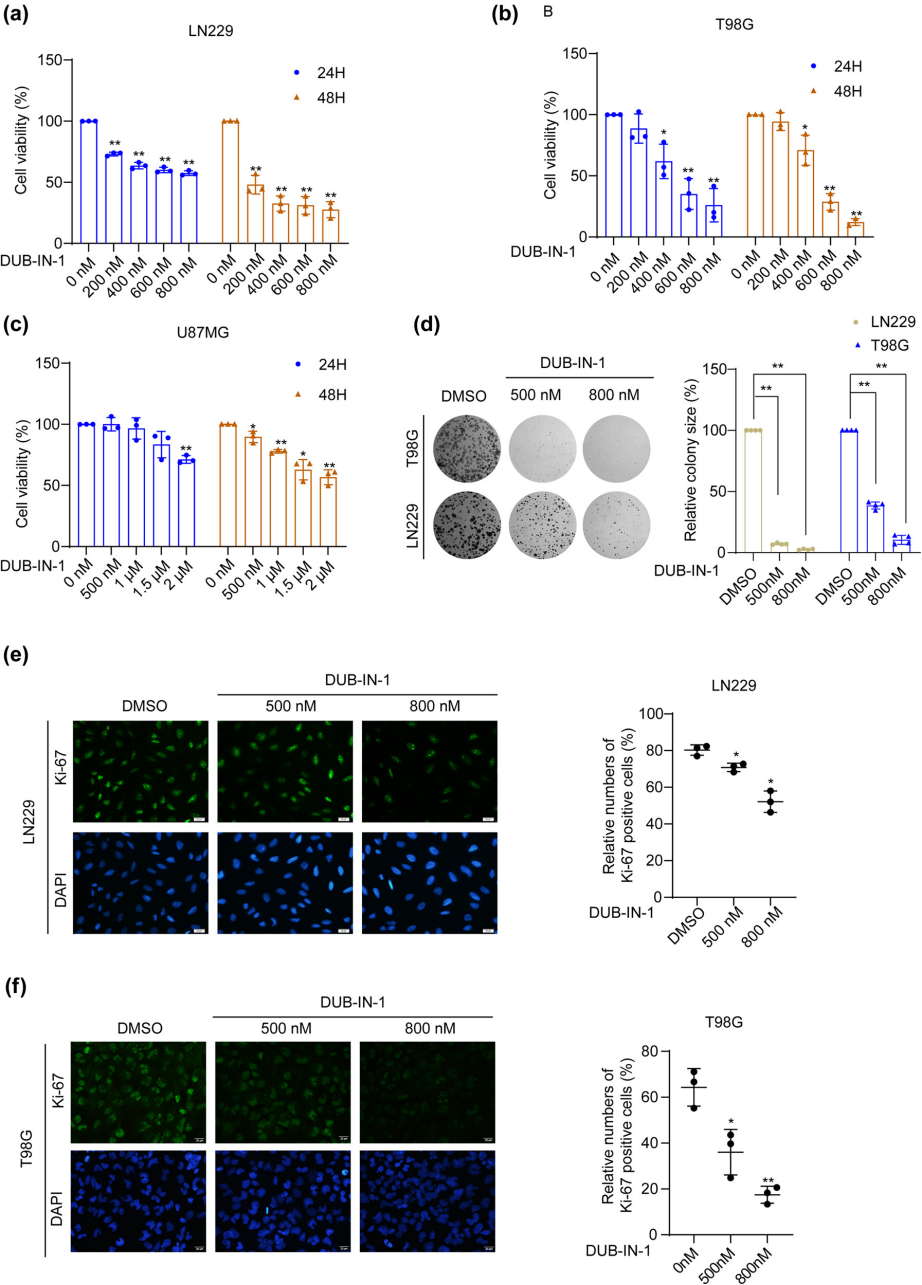
Pharmacological USP8 inhibition suppresses GBM cell proliferation. (a–c) LN229, T98G, and U87MG cells were treated with the USP8 inhibitor DUB-IN-1, and the cell viability was analyzed using the MTT assay. (d) Colony formation assays were performed on cells treated with the USP8 inhibitor DUB-IN-1. (e and f) The representative images of Ki-67 staining assay of T98G and LN229 cells treated with the USP8 inhibitor DUB-IN-1. Scale bar represents 20 µm. *P < 0.05, **P < 0.01 using Student’s *t*-test; *n* ≥ 3.

DUB-IN-1 was reported as a potent USP8 deubiquitinating activity inhibitor, with an IC_50_ of 0.85 µM for USP8 [13]. Using this inhibitor, we performed MTT assays to measure cell proliferation and observed a dose-dependent effect of USP8 inhibition on cell propagation in LN229, U87MG, and T98G cells (Figure 1a–c). As the U87MG cells appeared to be less sensitive to USP8 inhibition, we selected T98G and LN229 cell lines for subsequent experiments. Consistent with the results from the MTT assays, USP8 inhibition also led to marked reductions in colony formation by both T98G and LN229 cells (Figure 1d). We next examined the expression levels of the proliferation marker Ki-67 in LN229 and T98G cells with or without USP8 inhibition using immunofluorescence assays. As demonstrated in Figure 1e and f, the USP8 inhibitor DUB-IN-1 effectively reduced the percentages of cells with positive Ki-67 staining, suggesting a growth-suppressive influence of USP8 inhibition on these glioma cells. Collectively, these results from various proliferation assays consistently confirmed the inhibitory effects of the USP8 inhibitor DUB-IN-1, on the growth of GMB cells *in vitro*.

### 3.2 The migration of GBM cells is attenuated by the USP8 inhibitor DUB-IN-1

**Figure 2:**
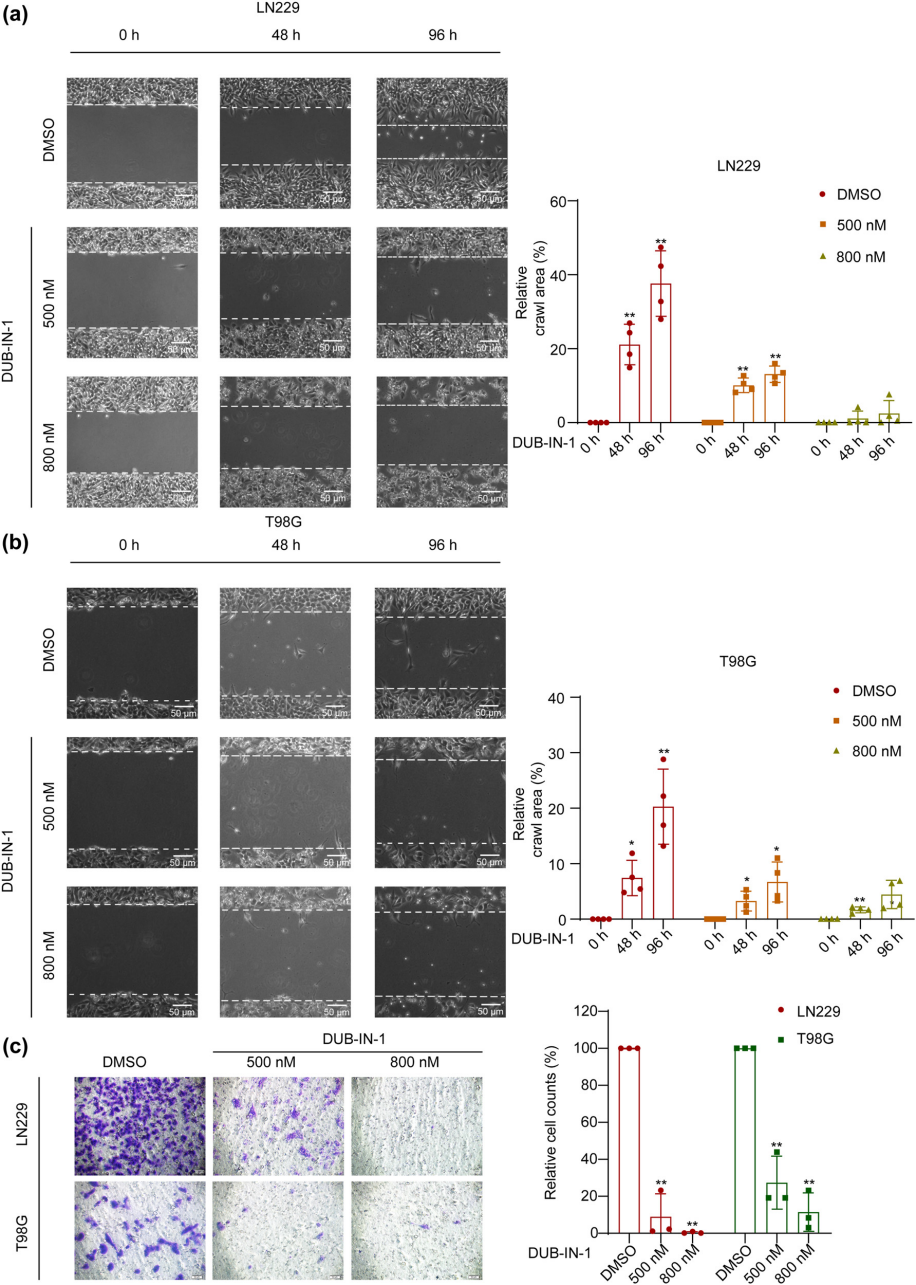
The migration of GBM cells is attenuated by DUB-IN-1. (a and b) The representative images of wound healing assay of T98G and LN229 cells treated with the USP8 inhibitor DUB-IN-1. White dotted lines indicate wound edges. Scale bar represents 50 µm. (c) The representative images of Transwell assay of T98G and LN229 cells treated with the USP8 inhibitor DUB-IN-1. Scale bar represents 50 µm. *P < 0.05, **P < 0.01 using Student’s *t*-test; *n* = 3.

The invasiveness of GBM cells presents a challenge for the complete surgical removal of tumor tissues [20,21]. Highly invasive GBM cells often infiltrate peritumoral normal brain tissues, thus making complete removal difficult and ultimately causing tumor recurrence that threatens the lives of the patient [21,22,23]. Therefore, we evaluated the impact of USP8 pharmacological inhibition on the migration of GBM cells. Wound healing assays showed that LN229 and T98G cells treated with the USP8 inhibitor DUB-IN-1 showed significantly decreased migration compared with the DMSO-treated control groups (Figure 2a and b). The effect of USP8 inhibition appeared stronger in LN229 cells, as the suppression of T98G cell migration required a higher concentration of the USP8 inhibitor DUB-IN-1 in T98G cells. We performed Transwell assays using LN229 and T98G cells to study the inhibitory effects of the USP8 inhibitor DUB-IN-1 on GBM cell migration. In accordance with results obtained from the wound healing experiments, the USP8 inhibitor DUB-IN-1 also markedly suppressed cell migration, as revealed through transwell assays with both LN229 and T98G cells (Figure 2c). Therefore, pharmacological USP8 inhibition demonstrated effective antimigration effects on GBM cells *in vitro*.

### 3.3 The USP8 inhibitor DUB-IN-1 decreases GBM stemness and sensitizes GBM cells to IR

**Figure 3:**
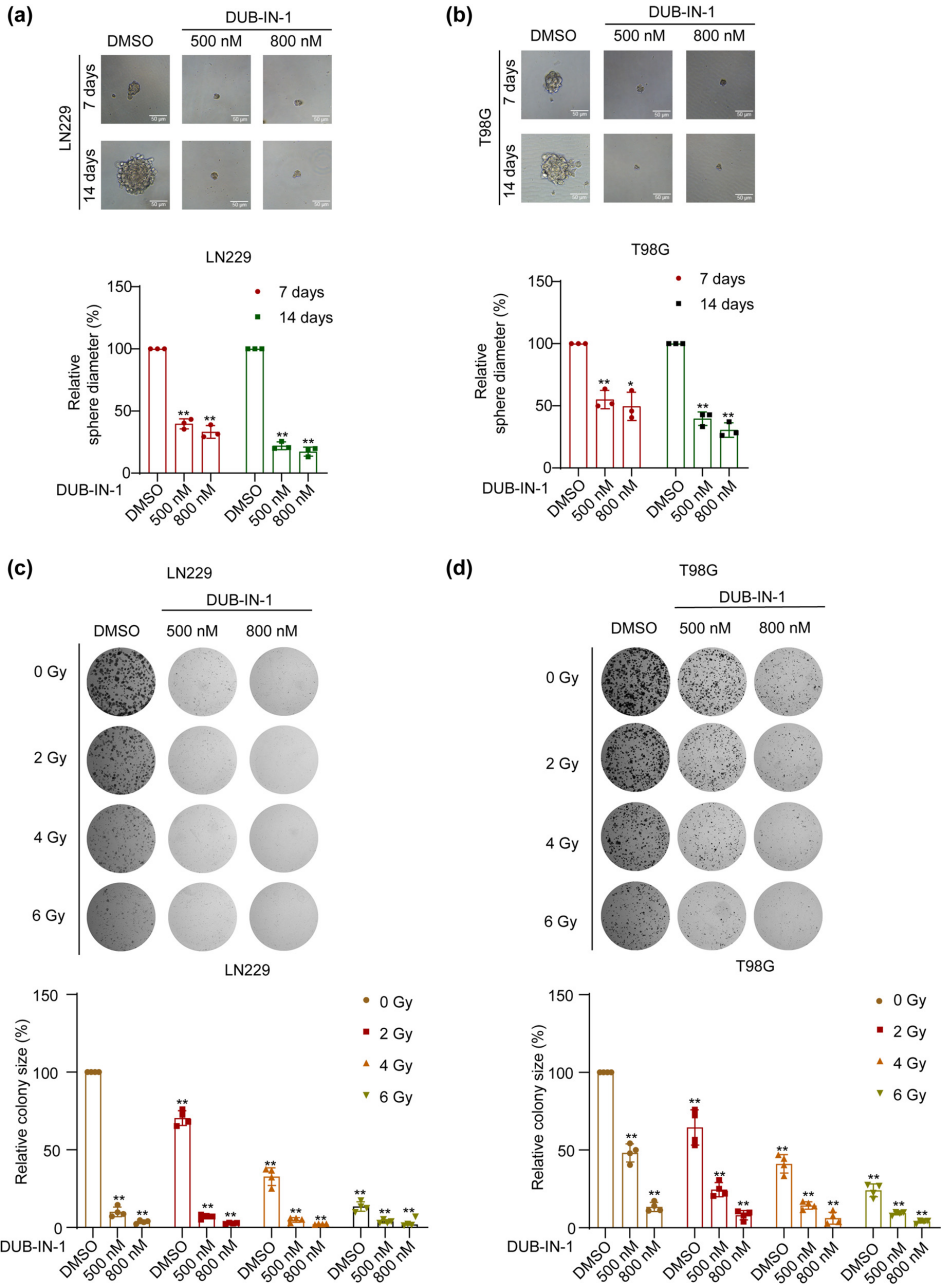
DUB-IN-1 decreases GBM stemness and sensitizes GBM cells to IR. (a and b) The representative images of spheroid formation assay of T98G and LN229 cells treated with the USP8 inhibitor DUB-IN-1. (c and d) After treatment with the USP8 inhibitor DUB-IN-1, a colony formation assay was performed to measure the proliferation of cells following the IR assay. *P < 0.05, **P < 0.01 using Student’s *t*-test; *n* ≥ 3.

GBM exhibits significant intratumoral heterogeneity, forming a range of tumor cell lineages with tumorigenic potential [24]. Accumulating evidence has established that GBM tissues often contain a substantial proportion of tumor stem cell subpopulations [25], which play vital roles in tumor initiation, malignant progression, drug resistance, radioresistance, and tumor recurrence [25,26,27]. Considering previous observations that USPs are involved in the stem cell properties of glioma, such as the association of USP22 with the stemness and tumorigenicity of glioma [28], and EPG5 deubiquitination through USP8 to maintain stemness [29], we hypothesized that the USP8 inhibitor DUB-IN-1 can possibly inhibit the stemness of glioma. To test this hypothesis, we conducted spheroid formation assays to investigate the stemness of GBM cells with or without USP8 inhibition. Cells treated with the USP8 inhibitor DUB-IN-1 showed significantly reduced spheroid size (Figure 3a and b), indicating a dramatic decrease in stemness. Radiotherapy is a major therapeutic modality for clinical management of GBM. To estimate the effect of USP8 inhibition on the radiosensitivity of GBM cells, we performed colony formation assays to evaluate the combined effects of IR and USP8 inhibition. As shown in Figure 3c and d, IR significantly decreased the number of colony clones formed by both LN229 and T98G cells in a dose-dependent manner, and this was significantly sensitized to irradiation-mediated suppresion mediated by the USP8 inhibitor DUB-IN-1.

### 3.4 The USP8 inhibitor DUB-IN-1 leads to decreased AURKA expression

**Figure 4:**
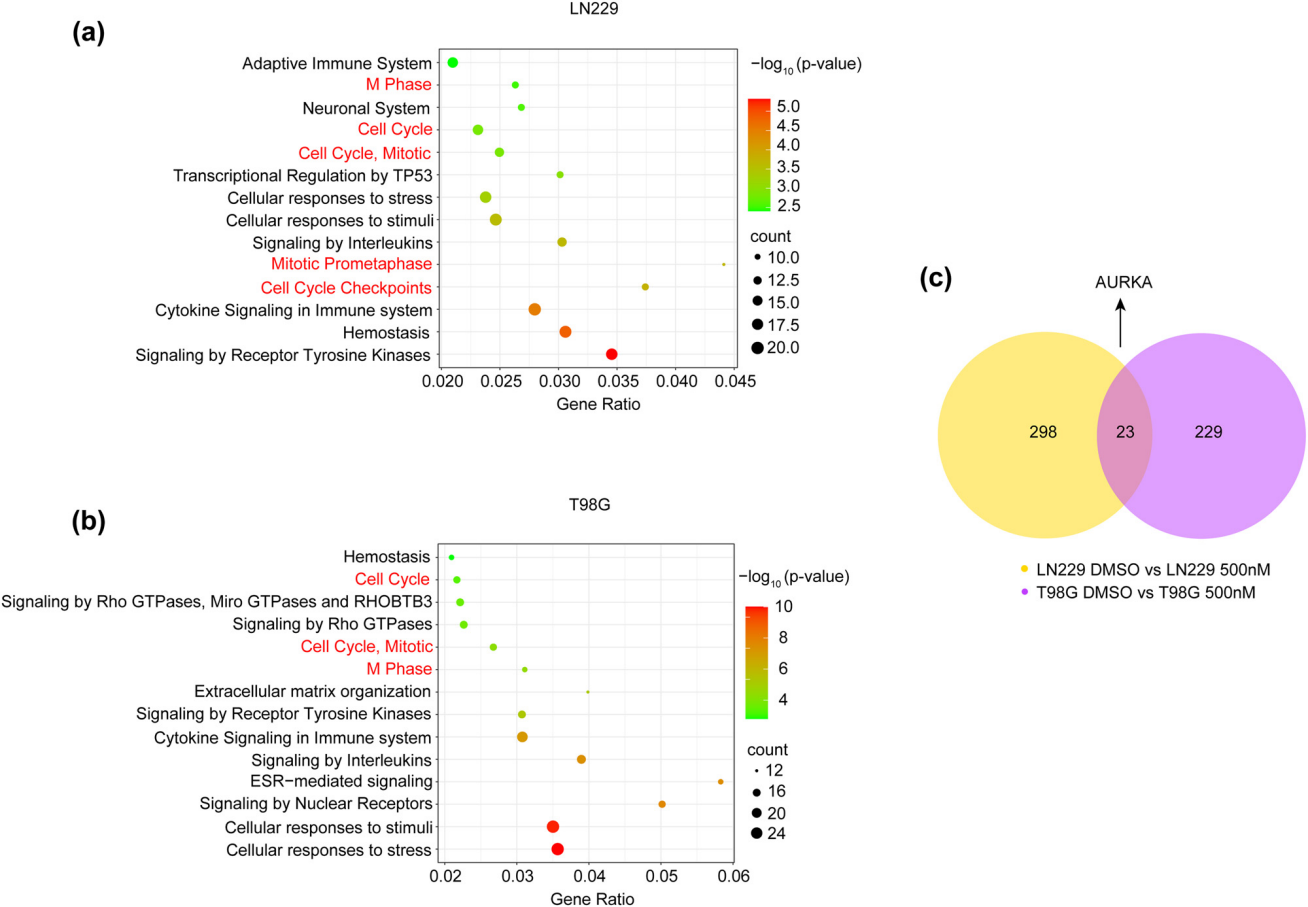
Transcriptome analysis of GBM cells treated with DUB-IN-1. (a and b) RNA sequencing data (LN229 DMSO vs LN229 500 nM and T98G DMSO vs T98G 500 nM) was analyzed using Metascape with reactome function enrichment analysis and the top 14 functional enrichments were selected, which were visualized using a bioinformatics online tool (www.bioinformatics.com.cn). (c) Venn diagram showing overlapping differentially gene numbers between LN229 vs T98G cells following treatment with the USP8 inhibitor DUB-IN-1 (*P* < 0.05, log_2_[fold change] > 1).

After confirming the inhibitory effects of pharmacological USP8 inhibition on glioma cell growth and stemness, we performed transcriptomic profiling of LN229 and T98G cells with or without USP8 inhibitor DUB-IN-1 treatment to investigate its underlying mechanisms. Differentially expressed genes following USP8 inhibitor DUB-IN-1 treatment in LN229 and T98G cells were analyzed using reactome enrichment. As demonstrated in Figure 4a and b, cell cycle-related functions were common in both LN229 and T98G cells in the top-listed reactome terms. These observations are consistent with our results of decreased cell growth following treatment with the USP8 inhibitor DUB-IN-1 in both GBM cell lines. Next, we investigated the common cell cycle-associated targets of USP8 inhibition in both LN229 and T98G cells. As shown in the Venn diagram in Figure 4c, differentially expressed genes were observed in both GBM cell lines following USP8 inhibition, and the expression of AURKA, which encodes a well-known cell cycle-related AURKA, was among the top 10 most significantly downregulated genes (Table 1).

**Table 1:** Top 10 overlapping down-regulated differentially expressed genes of LN229 and T98G cells

Gene name	LN229	T98G
HIST1H2AG	−2.189608365	−3.312903097
AC233968.1	−3.58089194	−3.231023759
PI15	−2.046971612	−3.138549889
KIF20A	−2.595581918	−2.526225595
PLK1	−1.962584231	−2.001025093
CENPE	−1.849243116	−1.83684878
AURKA	−1.593150497	−1.675818385
CENPA	−1.754749424	−1.550810046
NEK2	−1.552948615	−1.527897074
P2RX7	−3.146447522	−1.525680398

Table showing the top 10 overlapping down-regulated differentially genes set with log_2_ (fold change).

**Figure 5:**
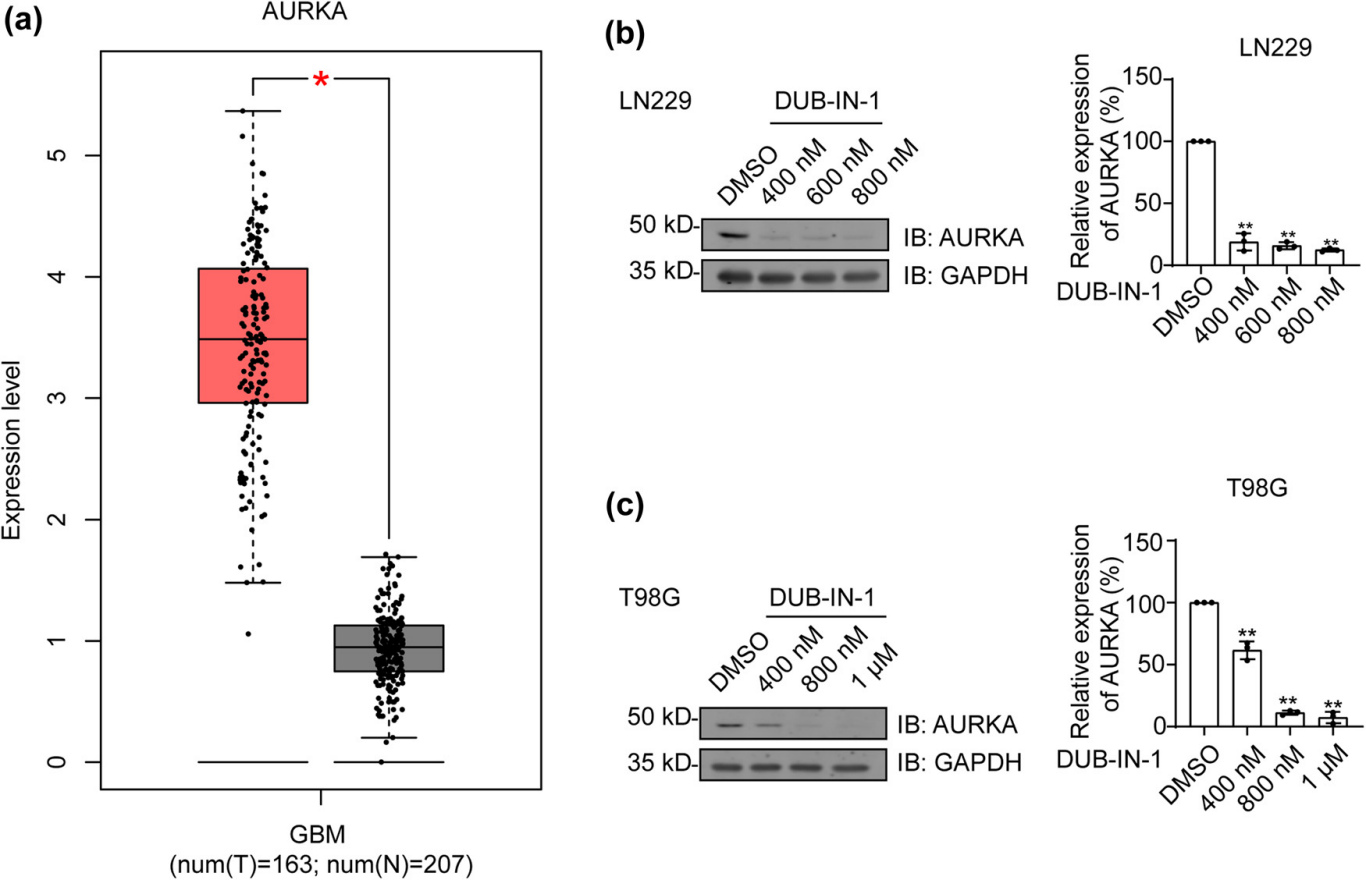
DUB-IN-1 leads to decreased AURKA expression. (a) Using GEPIA, the expression level of AURKA in tumor (T) tissues was shown to be significantly higher than that in nontumor (N) tissues, *P < 0.05. (b and c) The effects of different concentrations of the inhibitor on AURKA expression. *P < 0.05, **P < 0.01 using Student’s *t*-test; *n* ≥ 3.

Previous studies have shown elevated AURKA mRNA expression in glioma [30], and the protein levels of the AURKA were reported to be increased in glioma [31]. Using the Gene Expression Profiling Interactive Analysis (GEPIA) website, we compared the mRNA levels of AURKA in normal and GBM tumor tissues. In accordance with previous findings, AURKA expression was significantly increased in GBM tissues (Figure 5a). Next, we performed Western blotting experiments to validate the reduction of AURKA at the protein level. As shown in Figure 5b and c, AURKA protein expression was sensitive to the USP8 inhibitor DUB-IN-1 in a dose-dependent manner in both LN229 and T98G cells. In conclusion, our outcomes suggest that downregulation of AURKA expression likely contributes to the growth inhibition induced by USP8 pharmacological inhibition.

## 4 Discussion

Glioma typically originates from glial or precursor cells and accounts for approximately 30% of brain tumors as well as approximately 80% of malignant primary brain tumors [32], with more than 10,000 cases diagnosed yearly in the United States [33]. The survival time of patients with GBM has not been dramatically improved with current treatments. Therefore, further investigation of GBM tumorigenesis is necessary to identify high-quality biomarkers and potential therapeutic targets.

USP8 is a member of the USP family of DUBs and is closely associated with tumorigenesis and drug resistance. USP8 has also been proposed as a prognostic factor for breast cancer [34]. Previous studies have revealed various functions of USP8 in various tumors [12,35,36]. The current study focused on its role in GBM cell growth, migration, and stemness. Members of the USP family have been reported to regulate GBM tumorigenesis [37,38,39]. USP15 promotes tumor cell invasion and proliferation in GBM [40]. Regarding USP8, its tumor-promoting functions have been reported in several cancer types, and its inhibition reduces the invasion of cholangiocarcinoma cells [41]. DUB-IN-1 treatment inhibited esophageal squamous cell carcinoma cell growth and induce G2/M cell cycle arrest, apoptosis, and autophagy through DNA damage-induced p53 activation [42]. However, there are few studies on USP8 in GBM. Therefore, we studied the effect of DUB-IN-1 on GBM cells. U87MG cells were found to have a lower sensitivity to DUB-IN-1 (Figure 1a–c). In the cell viability assay, the inhibition rate of U87MG cells was lower than that of the previous two cell types when the DUB-IN-1 dose was thrice applied to T98G and LN229 cells. Therefore, LN229 and T98G cells were selected for the follow-up study. In these two cell lines, DUB-IN-1 significantly inhibited the formation of cell colonies (Figure 1d). In addition, DUB-IN-1 effectively reduced the percentage of Ki-67-positive cells in LN229 and T98G cells, as shown using an immunofluorescence assay (Figure 1e and f). In summary, DUB-IN-1 inhibited the growth of GBM cells. Highly invasive GBM cells often infiltrate the normal brain tissue around the tumor [21], making it difficult to completely remove the tumor tissue [23] and ultimately leading to tumor recurrence [21,22]. Studies have shown that USP8 can promote migration and invasion in tumors. In this study, the effects of DUB-IN-1 on GBM LN229 and T98G cell migration were evaluated using wound healing and Transwell assays. The results showed that DUB-IN-1 effectively inhibited the migration and invasion of LN229 and T98G cells (Figure 2). From a pharmacological perspective, USP8 inhibition had an effective anti-migration effect on GBM cells *in vitro*. It is noteworthy that GBM stemness is a major cause of relapse and treatment resistance [43,44]. The involvement of deubiquitylases in maintaining cancer cell stemness has been previously reported [45,46]. The inhibition of USP1 can inhibit the growth of GBM cells by inhibiting stem cell renewal and radioresistance [47]. SMO enhances the radiation resistance of GBM cells by promoting the transcription of USP3 and activating Claspin-dependent ATR-Chk1 signaling [48]. Our study showed that the stemness of LN229 and T98G cells was significantly attenuated by the USP8 inhibitor DUB-IN-1, as revealed by the results of the spheroid formation assay (Figure 3a and b). In addition, we evaluated the sensitivity of LN229 and T98G cells treated with DUB-IN-1 to IR using a colony formation assay. The experimental results showed that DUB-IN-1 treatment effectively sensitized LN229 and T98G cells to IR (Figure 3c and d).

To investigate the potential mechanism by which DUB-IN-1 inhibits the growth, migration, and stemness of LN229 and T98G cells, transcriptome analysis was performed on LN229 and T98G cells, and differentially expressed genes in LN229 and T98G cells were analyzed using reactome functional enrichment. The results showed that the first several pathways are related to cell cycle function. Based on the Venn diagram, differentially expressed genes were found in both LN229 and T98G cells after treatment with DUB-IN-1 (Figure 4c), and the common differentially expressed genes were analyzed. AURKA, a gene associated with the cell cycle, was among the 10 most significantly downregulated genes, and the transcriptome results showed that AURKA expression decreased after the use of the inhibitor, which was consistent with our previous findings. DUB-IN-1 treatment has been reported to induce G2/M cell cycle arrest by upregulating the protein level of p21 and trigger apoptosis by modulating p53 target proteins, including Bax, Noxa, and Puma [42]. AURKA may act as a potential mechanism through which USP8 inhibitors inhibit the growth, migration, and stemness of LN229 and T98G cells.

AURKA is located on chromosome 20q13.2 and plays a crucial role in the control of mitotic progression. The AURKA oncogene is amplified in various tumors, including glioma [31]. It has been reported that the expression of AURKA mRNA is increased in glioma [30], which is consistent with our results from bioinformatic analysis (Figure 5a). Interestingly, the USP8 inhibitor DUB-IN-1 decreased AURKA mRNA expression and also inhibited AURKA expression in a dose-dependent manner (Figure 5b and c). It has been shown that the AURKA activity is vital to promote colony formation and tumor growth [49]. In addition, inhibition of AURKA was reported to improve the efficacy of radiotherapy [50]. Therefore, the reduction in AURKA expression induced by pharmacological USP8 inhibition is likely a significant factor in GBM cell suppression. However, this study lacks *in vivo* analysis to verify the *in vitro* studies, and other research methods are required for further study in the future [14,51,52,53,54].

In summary, by demonstrating the effectiveness of pharmacological USP8 inhibition in suppressing GBM cell growth, migration, and stemness, as well as in promoting the sensitivity of GBM cells to radiotherapy, our findings suggest USP8 as a potential therapeutic target for the treatment of GBM, warranting further investigation.
